# Mechanisms of Linezolid Resistance in Mycobacteria

**DOI:** 10.3390/ph16060784

**Published:** 2023-05-24

**Authors:** Wei Chong Gan, Hien Fuh Ng, Yun Fong Ngeow

**Affiliations:** Dr. Wu Lien-Teh Centre for Research in Communicable Diseases, M. Kandiah Faculty of Medicine and Health Sciences, Universiti Tunku Abdul Rahman, Jalan Sungai Long, Bandar Sungai Long, Kajang 43000, Selangor, Malaysia; wchongg@yahoo.com (W.C.G.); hfng@utar.edu.my (H.F.N.)

**Keywords:** *Mycobacterium tuberculosis*, non-tuberculous mycobacteria, *Mycobacteroides abscessus*, linezolid resistance, genetic determinants

## Abstract

Mycobacteria form some of the most notorious and difficult-to-treat bacterial pathogens. As a group, they are intrinsically resistant to many commonly used antibiotics, such as tetracyclines and beta-lactams. In addition to intrinsic resistances, acquired multidrug resistance has also been observed and documented in *Mycobacterium tuberculosis* (MTB)*, Mycobacterium leprae* and non-tuberculous mycobacteria (NTM). To combat multidrug resistant infections by these pathogens, innovative antimicrobials and treatment regimens are required. In this regard, linezolid, an oxazolidinone introduced for clinical use just two decades ago, was added to the therapeutic armamentarium for drug-resistant mycobacteria. It exhibits antibacterial activity by binding to the 50S ribosomal subunit and inhibiting protein synthesis. Unfortunately, linezolid resistance has now been documented in MTB and NTM, in many parts of the world. Most linezolid-resistant mycobacterial strains show mutations in the ribosome or related genes, such as in the *rplC, rrl* and *tsnR* genes. Non-ribosomal mechanisms appear to be rare. One such mechanism was associated with a mutation in *fadD32*, which encodes a protein that plays an important role in mycolic acid synthesis. Mycobacterial efflux proteins have also been implicated in linezolid resistance. This review summarises current knowledge of genetic determinants of linezolid resistance in mycobacteria, with the aim of contributing information that could facilitate the discovery of new therapeutic approaches to overcome, delay or avoid further developments of drug resistance among these important pathogens.

## 1. Introduction

### 1.1. Drug Resistance in Mycobacteria

The genus *Mycobacterium* incudes well-established pathogens, such as *Mycobacterium tuberculosis* (MTB), the cause of tuberculosis (TB), *Mycobacterium leprae* and *Mycobacterium lepromatosis,* the causative agents of leprosy, as well as more than 1800 species of NTM, many of which are opportunistic pathogens associated with a wide spectrum of skin, soft tissue and deep organ infections, especially among the immunocompromised [1]. The morbidity and mortality resulting from mycobacterial infections are significant, and recovery is heavily dependent on effective antimicrobial therapy.

Unfortunately, mycobacteria are intrinsically resistant to many antibiotics [2,3]. This is due in part to the mycolic acids in their cell walls as well as the presence of various proteins that promote antibiotic resistance, such as ribosomal protection proteins and efflux pumps [4,5]. In addition, there has been an increasing number of reports on acquired resistance against previously effective antibiotics, including tigecycline, fluoroquinolones and streptomycin [6,7,8,9,10,11,12], following extensive and possibly inappropriate antibiotic use.

Treating TB requires the use of drug combinations over long periods. The standard initial treatment regimen for tuberculosis has been the administration of four drugs (isoniazid, rifampicin, pyrazinamide and ethambutol) together for 4 to 6 months. However, incidents of MTB infections resistant to these antibacterials have been reported for decades [13]. In 2021, the World Health Organisation (WHO) estimated around 450,000 people to be infected with multidrug-resistant tuberculosis (MDR-TB, resistant to rifampicin and isoniazid) worldwide [14]. XDR-TB (extensively drug-resistant TB), originally defined by its resistance to isoniazid, rifampicin, plus any fluoroquinolone and at least one aminoglycoside/aminocyclitol, was also detected in multiple countries between the years 2000 to 2004, forming an average of about 10% of MDR-TB isolates [15]. In January 2021, WHO redefined XDR-TB as TB caused by MDR-TB strains which are also resistant to any fluoroquinolone and at least one additional Group A drug (levofloxacin, moxifloxacin, bedaquiline and linezolid) [16].

Leprosy is one of the oldest infectious diseases, first reported more than 2000 years ago. It is caused by *M. leprae* and *M. lepromatosis.* The two species share 92% nucleotide identity and can be co-infectants in a patient with leprosy [17,18,19]. Despite worldwide elimination programmes and use of multidrug treatment under the guidance of WHO in the past four decades, this devastating infection persists, particularly in less affluent populations. As can be expected for protracted infections with poor treatment compliance, drug resistance appeared and spread to cause relapses and therapeutic problems [20,21]. To delay the emergence of resistance, WHO recommended combination therapy with different combinations of rifampicin, dapsone, fluoroquinolones, tetracyclines, macrolides, clofazimine and bedaquiline, for different clinical presentations of leprosy [21]. Linezolid was also found to be bactericidal against rapidly multiplying *M. leprae*. Unfortunately, it is unsuitable for routine therapy as prolonged use of this antibacterial has been associated with severe side effects in a large proportion of patients [22]. 

Drug resistance among the NTM has been reported for both slow and rapid-growing species associated with human infections. The mainstay of treatment for most NTM infections consists of a macrolide (clarithromycin or azithromycin) together with either an aminoglycoside, cefoxitin, imipenem or tigecycline for fast growers [23] or ethambutol and rifampicin for slow growers [24]. As resistance has appeared to all these drugs and treatment failures are frequent [25], the discovery of new and more efficient therapies against NTM is important and urgent.

### 1.2. Linezolid

Linezolid is a member of the oxazolidinones, which are fully synthetic drugs that inhibit protein synthesis in bacteria. It was discovered in 1996 but only approved for clinical use in the year 2000 [26]. 

#### 1.2.1. Mechanism of Action

Initially, it was proposed that linezolid acts by binding to the 23S rRNA of the 50S ribosomal subunit, near the peptidyltransferase centre (PTC) of the would-be-ribosome, before the formation of the 70S initiation complex [27]. Upon binding to the subunit, the antibiotic would inhibit the ability of the 50S ribosomal subunit to bind to the 30S subunit, which would prevent the formation of the 70S initiation complex (consisting of the 50S and 30S subunits, and the fMet-tRNA and mRNA), obstructing the early stages of protein synthesis. This proposed mechanism of action is unique and unlike other antibiotics that inhibit the elongation part of protein synthesis. 

However, later studies and crystallography analyses suggested that it is more likely that linezolid binds to the PTC of the ribosome, overlapping with the binding sites of antibiotics such as clarithromycin and clindamycin [28]. In addition to this, it was found that linezolid binds better to the A site of the ribosome when alanine is the penultimate amino acid in the nascent chain [29]. This is because the alanine residue would fit into a hydrophobic pocket within a structure of the linezolid, helping it to bind to the ribosome and act as an obstruction within the PTC, sterically interfering with the formation of the peptide bond between the tRNA in the A and E sites.

#### 1.2.2. Spectrum of Activity

Linezolid is active against both aerobic and anaerobic Gram-positive bacteria [30,31,32]. Among the Gram-negative bacteria, linezolid showed activity against anaerobes, such as *Bacteroides*, *Prevotella*, *Fusobacterium*, *Porphyromonas* and *Veillonella* species [31], but is largely ineffective against Gram-negative aerobes, except for some *Pasteurella* and *Moraxella* species [32]. Its inactivity against most Gram-negative aerobes is presumed to be due to its expulsion by endogenous efflux pumps in these bacteria [33]. Recently, however, it was shown that it is possible to enlarge the activity spectrum of this drug to cover more Gram-negative bacteria with the use of silica xerogel as a drug carrier [34].

#### 1.2.3. Clinical Use

Linezolid is often used as a last-resort drug for infections caused by drug-resistant Gram-positive bacteria, such as methicillin-resistant *Staphylococcus aureus*, penicillin and macrolide-resistant pneumococci and vancomycin-resistant enterococci with VanA, VanB, or VanC resistance determinants [32]. Against mycobacterial diseases, it has been recommended for the treatment of NTM infections [35], including refractory cases of disseminated infections [36]. It has also been classified by the WHO as a Class A drug for the treatment of MDR-TB and XDR-TB in humans [37,38]. Conradie et al. (2020) reported 90% favourable outcomes at 6 months after treatment of XDR-TB and MDR-TB patients with a combination of bedaquiline, pretomanid and linezolid [39]. In this multidrug regimen, the efficacy of linezolid is boosted by bedaquiline, which inhibits the mycobacterial proton pump that is responsible for drug efflux, and pretomanid, which inhibits mycolic acid synthesis, resulting in a less hydrophobic cell wall that allows easier entry for hydrophilic drugs such as linezolid. Synergism has also been observed with other ribosome-targeting antibiotics, such as clarithromycin and capreomycin [40,41]. Interestingly, linezolid has been reported to have an antagonistic effect with first-line drugs for TB, such as isoniazid and pyrazinamide, in murine models [42]. The reason behind this antagonism has been hypothesised to be due to linezolid’s effect on the absorption of first-line drugs, but the exact antagonistic mechanism and interaction between these antibiotics are still not well understood.

### 1.3. Linezolid Resistance in Mycobacteria

Just 7 years after linezolid was approved for clinical use, the first case of linezolid-resistant tuberculosis was reported in Germany [43]. In recent years, studies across the United States of America, Europe, and Asia have shown a resistance rate of 4.2% among MTB clinical isolates [44]. Wasserman et al. (2019) reported that a third of patients (13/39) from a high-risk cohort in South Africa with linezolid-based treatment failure harboured linezolid-resistant MTB [45]. Among the factors investigated, they found the number of anti-TB drugs taken by patients to be significantly associated with the risk of developing linezolid-resistant TB (with the resistant group taking a median of 10 drugs compared to eight in the non-resistant group). In a study from India, linezolid-resistant MTB was found in about 6.7% (23/343) of patients diagnosed with MDR-TB [46]. Researchers from Moscow reported the emergence of linezolid-resistant strains from TB patients undergoing treatment with Group A drugs that included linezolid [47]. In their cohort of XDR-TB patients, 6.21% (20/322) were found to have linezolid-resistant MTB.

Linezolid resistance has also been reported in NTM. Among patients in the United Kingdom, resistance had been detected in 51.7% (30/58) and 54.2% (13/24) of *M. abscessus* and *M. chelonae* isolates, respectively [48]. In China, Ye et al. (2019) reported 43.8% resistance among 194 *M. abscessus* isolates [49]. Similarly, reports from Taiwan indicated a high prevalence of resistance with rates of 24.6% (17/69) among *M. fortuitum*, 5.1% (2/39) among *M. chelonae* and 42.4% (39/92) among *M. abscessus* [50]. However, Tu et al. (2022) found widely discordant resistance rates using different methods of testing. With broth microdilution, they detected resistance in 45.5% (10/22) of *M. abscessus* isolates and 12.5% (1/8) of *M. fortuitum* isolates. However, with the Etest (not endorsed by the European Committee on Antimicrobial Susceptibility Testing and Clinical and Laboratory Standards Institute), the resistance rates for the same isolates increased to 90.9% for *M. abscessus* and 70.0% for *M. fortuitum* [51], a stark reminder of how MIC results could be affected by the method used to determine them. 

It is now evident that linezolid-resistant mycobacteria are an emerging problem all over the world, and efforts have to be undertaken to better understand and manage these infections so that they can be more adequately contained.

## 2. Mechanisms of Linezolid Resistance in Mycobacteria

**Figure 1:**
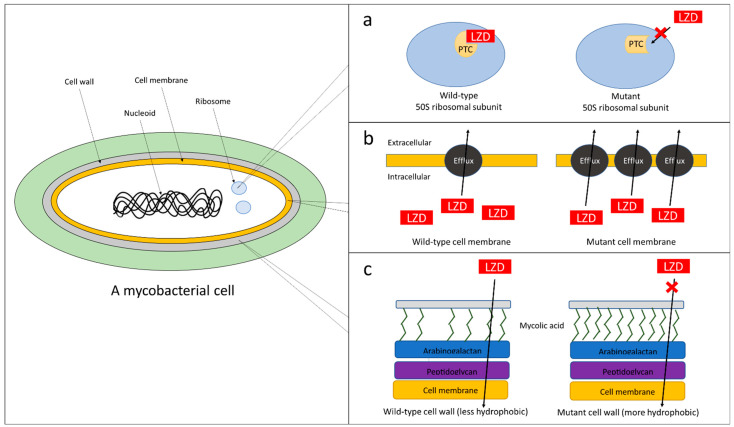
Mechanisms of linezolid resistance reported in mycobacteria. The *tsnR* loss-of-function mutations are not shown in this figure due to a lack of information on the proposed mechanism related to these mutations. (**a**) Ribosomal changes (mutations in either 23S rRNA or L3 ribosomal protein) that alter the conformation of the rRNA at the binding site of linezolid, making it unable to bind to the peptidyltransferase centre (PTC). (**b**) Mutations leading to the over-expression of efflux pumps that remove intracellular linezolid molecules. (**c**) The mutation in FadD32 that is involved in the synthesis of mycolic acid. This mutation may lead to the production of more mycolic acids, increasing the hydrophobicity of the mycobacterial cell wall and reducing the intake of linezolid (a hydrophilic drug). Red crosses indicate the reduced activity of linezolid.

Table 1 and Figure 1 summarise the linezolid resistance mechanisms that have been reported for mycobacteria.

### 2.1. Ribosomal Mechanisms

In mycobacteria, as in other Gram-positive pathogens such as staphylococci and enterococci, the most frequently reported mechanism of linezolid resistance is a mutation in the ribosome structure near the PTC. Mutations in or near the PTC would result in linezolid being unable to bind to the ribosome (Figure 1a), thus preventing the normal process of protein synthesis. These ribosomal mutations are commonly found in the *rplC* gene, the 23S rDNA, and genes encoding ribosomal enzymes, such as the methyltransferase [57].

#### 2.1.1. Mutations in *rplC*

The *rplC* gene encodes the ribosomal protein L3, a constituent of the 50S ribosomal subunit that is involved in the PTC of the ribosome. In MTB, the most frequent determinants of linezolid resistance are mutations in this gene [53]. The dominant genetic determinant was found to be a point mutation, t460c, that alters cysteine at position 154 to arginine. Makafe et al. (2016) used gene-overexpression to study the effects of this mutation in MTB [58] and found that overexpressing the mutated *rplC* gene increased the linezolid minimum inhibitory concentration (MIC) four-fold (from 1 mg/L to 4 mg/L). They also showed this increase in MIC to be dependent on the activity of the promoter, as linezolid resistance was exhibited when *rplC* was cloned downstream of a strong promoter but not downstream of a weaker promoter.

The Cys154Arg mutation is thought to function similarly to the Asn149Arg mutation in the *Escherichia coli* L3 protein [59]. The Asn149 residue in the ribosome of *E. coli* corresponds to the location of the Cys154 residue in MTB, and the Asn149Arg mutation in *E. coli* was also linked with linezolid resistance. Computer modulations showed that the amino-acid change at this location would affect interactions with other close-by residues, resulting in slight conformational changes in the L3 protein which might inhibit the ability of linezolid to bind to the PTC region. However, these interactions have not been studied in depth in mycobacteria. Further investigations are needed to confirm their involvement in linezolid resistance in mycobacteria.

#### 2.1.2. Mutations in 23S rRNA (*rrl*)

The 23S rRNA is an important component of the ribosome and forms a large part of the 50S ribosomal subunit. It forms the PTC, is centred around Domain V of the rRNA, and is one of the most targeted binding sites for ribosome-targeting antibiotics such as chloramphenicol [60]. As such, mutations to the 23S rRNA are also quite frequently observed in linezolid-resistant mycobacteria [61]. Mutations in the *rrl* gene, which encodes the 23S rRNA, have been observed in many clinical isolates, particularly g2814t, which has been reported in many locations as far apart as India, South Africa, and Russia [45,46,47,52].

Linezolid resistance-conferring mutations in the 23S rRNA have been found to be mostly concentrated in its two domains, Domain V and Domain VI [62]. The effect of mutations in Domain V on linezolid resistance should be expected, as this domain forms the PTC of the ribosome, a site that linezolid binds to. However, it is not well understood how mutations in Domain VI confer linezolid resistance on mycobacteria. One possible mechanism is that mutations affect the interactions between the domain and the L6 ribosomal protein, which is an important protein that is located near the binding site of the PTC [63]. Furthermore, there are cross-linkages between the helixes of Domain VI, the L6 protein, and Domain V, which are important in maintaining the secondary structure of the ribosome. As such, mutations in Domain VI may affect the structure of the rRNA in a manner that confers linezolid resistance [64]. More investigations are required to further understand the link between these ribosomal structures and linezolid resistance.

Ng and Ngeow (2023) observed mutations in the 23S rRNA that conferred a high level of linezolid resistance on *M. abscessus* [54]. They selected two stepwise mutants: the first-step mutant carried a mutation in the *fadD32* gene that is involved in mycolic acid synthesis, and the second-step mutant accumulated more mutations in the 23S rRNA (g2244t and g2788t). In the second-step mutant, these 23S rRNA mutations apparently increased the linezolid MIC to >256 mg/L from 0.25 mg/L in the wild-type strain and 1 mg/L in the first-step mutant. They correspond to the mutations (g2270t and g2814t, found in Domain V [61]) in MTB that are the most prevalent 23S rRNA mutations in linezolid-resistant mutant strains [65]. However, the level of resistance in MTB is generally much lower than that observed in *M. abscessus* by Ng and Ngeow (2023) in their study [54]. Furthermore, in MTB, these mutations have so far been observed only separately and not together in the same strain. It is possible that the two mutations work in tandem to achieve a high level of linezolid resistance in *M. abscessus*.

#### 2.1.3. *tsnR* Loss of Function

In an analysis of clinical MTB strains, Li et al. (2021) observed that loss-of-function mutations in the putative *tsnR* gene occurred in some of these strains [55,66]. The *tsnR* gene hypothetically encodes the 23S rRNA methyltransferase in MTB. In their study conducted using the CRISPRi system, Li et al. found that the knockdown of *tsnR* conferred resistance to linezolid. Their observations concurred with those of a study in *S. aureus*, in which *tsnR*-knockout mutants also exhibited increased resistance to linezolid [67].

However, while a link between *tsnR* and linezolid resistance has been observed, the gene itself and its functions are still not well understood in MTB. Furthermore, the exact mechanism as to how the decreased expression or loss of function of *tsnR* confers linezolid resistance is still not clear. In many cases, such as in *Streptomyces* spp., methyltransferases confer resistance to ribosome-targeting antibiotics through the addition of a methyl group to the 23S rRNA, which would sterically interfere with the binding of the antibiotics [68]. This appears to be the opposite of the relationship between the *tsnR* loss of function and linezolid resistance observed in MTB and *S. aureus*.

### 2.2. Non-Ribosomal Mechanisms

#### 2.2.1. Efflux

In recent years, gene mutations leading to the increased translation of genes responsible for uptake and efflux have been reported to be the cause of linezolid resistance in previously linezolid-susceptible strains of mycobacteria (Figure 1b). Srivastava et al. (2017) found single-nucleotide variants in genes encoding efflux pump/transporters (Rv0545c, Rv0930, Rv2477 and Rv3331) and a transcriptional regulator Rv0890c in MTB isolates from patients who failed linezolid-based treatment [56]. Furthermore, the addition of efflux pump inhibitors, thioridazine or reserpine, reduced the MIC of these MTB isolates from 1 mg/L to 0.25 mg/L. Sander et al. (2002) considered it likely that linezolid resistance in *M. smegmatis* mutants with non-ribosomal mutations is caused by either decreased drug uptake into the bacterial cell or increased active efflux of the drug, caused, for example, by altering the specificity or activity of an efflux transporter [69]. Using whole-genome sequencing, Ye et al. (2019) sequenced 194 *M. abscessus* isolates and found that only 8.2% of linezolid-resistant strains harboured mutations in the 23S rRNA. Upon performing RT-qPCR assays to determine the up-regulated genes responsible for the resistances, it was found that there were higher transcriptional levels of the efflux pumps *lmrS* and *mmpL9* [49].

#### 2.2.2. Mutations in *fadD32*

Ng and Ngeow (2023) observed that a mutation, c880t (His294Tyr), at the *fadD32* gene in *M. abscessus* conferred linezolid resistance to the mutant, increasing the MIC fourfold (0.25 mg/L to 1 mg/L) [54]. Aside from linezolid, the mutant also demonstrated cross-resistance to other hydrophilic antibiotics such as imipenem and vancomycin. These observations are reminiscent of the findings of Carroll et al. (2011), who demonstrated increased susceptibility to ethambutol (2 mg/L to 1 mg/L) and ampicillin (>32 mg/L to 1 mg/L), both of which are hydrophilic antibiotics, in a *fadD32*-knockdown MTB mutant [70]. The *fadD32* gene is a highly conserved gene in mycobacteria that encodes for a fatty acyl-AMP ligase, which plays a role in the biosynthesis of mycolic acid, a major constituent of the mycobacterial cell wall [71]. In mycobacteria, mycolic acid in the cell wall is known to reduce the permeability of the cell wall towards hydrophilic substances by creating a hydrophobic, waxy shield [72]. Thus, the c880t mutation in *fadD32* could have led to linezolid resistance by increasing mycolic acid synthesis, leading to the creation of a more hydrophobic cell wall which inhibited the entry of hydrophilic antibiotics (Figure 1c).

## 3. Conclusions and Future Perspectives

In general, most of the mutations responsible for linezolid resistance in mycobacteria are found at the binding site of linezolid, involving structures immediately adjacent to the PTC area of the ribosome, such as the L3 protein and Domain V of the 23S rRNA, as well as structures close by, such as Domain VI. These mutations, being the most prevalent, are the most studied determinants of linezolid resistance in mycobacteria. The mutation in the *fadD32* gene represents a resistant determinant that is not directly related to the structure of the rRNA. The FadD32 protein is an essential protein involved in the synthesis of mycolic acid, the component that gives the mycobacterial cell wall its signature waxy, hydrophobic characteristic. The exact nature of the mutation, its direct effect on the cell wall, and its interactions with hydrophilic antibiotics have not yet been studied in depth. Another gene in mycobacteria that is still largely unstudied for its role in linezolid resistance is *tsnR*, which encodes a 23S rRNA methyltransferase. Its implications in protein synthesis disruption and possible interactions with other ribosome-targeting antibiotics await further elucidation.

Currently, most of the studies concerning linezolid resistance in mycobacteria focus on MTB, which is understandable owing to its being the flagship pathogen among mycobacteria. However, many NTM species among both slow and rapid growers also exhibit multidrug-resistance phenotypes [35,49,54], and subspecies such as those of *M. abscessus* can show different antibiotic susceptibilities [73]. Hence, future endeavours should be made to better understand the prevalence and mechanisms of linezolid resistance across different NTM species and subspecies.

Horizontal transfer of the linezolid resistance gene, *cfr* (encoding a 23S rRNA methyltransferase), has been reported in Gram-positive organisms, such as staphylococci and enterococci [74]. While horizontal gene transfer has rarely been reported in mycobacteria, some, such as *M. canettii* and *M. smegmatis*, have exhibited the ability to acquire genes via lateral DNA transfer [75,76,77]. Therefore, another area for future studies could be the horizontal gene transfer of linezolid resistance, which has yet to be reported for mycobacteria.

Linezolid is an important drug for the treatment of multidrug-resistant mycobacteria. A thorough understanding of resistance mechanisms and efficient monitoring of drug resistance are essential for improved clinical and public health management. The inclusion of new gene targets for routine drug susceptibility testing will lead to the rapid detection of linezolid-resistant strains and the selection of appropriate therapy. The designing of new drugs or drug combinations based on resistance mechanisms can help to curb the global spread of linezolid resistance.

## Figures and Tables

**Table 1:** Linezolid resistance determinants reported in mycobacteria.

Reference	Source	Nation	Mycobacterial Species	Gene(s)	Mutation(s)	MIC (mg/L)
Wasserman et al., 2019 [45]	Clinical strains	South Africa	*M. tuberculosis*	*rrl*	g2814t, g2270t	2–8
				*rplC*	t460c, g546a	4–8
Ushtanit et al., 2021 [47]	Clinical strains	Russia	*M. tuberculosis*	*rrl*	g2270t, a2801c, g2714t	1–4
				*rplC*	t460c	2–16
Nambiar et al., 2021 [52]	Clinical strains	India	*M. tuberculosis*	*rrl*	g2814t	>1
				*rplC*	Cys154Arg ^	
Beckert et al., 2012 [53]	Laboratory-derived mutants and clinical strains	Germany	*M. tuberculosis*	*rplC*	t460c	4–16
Ye et al., 2019 [49]	Clinical strains	China	*M. abscessus*	*rrl*	g15a, t328c, g348a, c1445t, c1582a, t2138c, a2271c, c2432t, g3048a	≥32
Ng and Ngeow, 2023 [54]	Laboratory-derived mutants	Malaysia	*M. abscessus*	*fadD32*	c880t	1
				*rrl*	g2244t and g2788t *	>256
Li et al., 2021 [55]	Clinical isolates (in silico) ^#^	Various countries	*M. tuberculosis*	*tsnR*	Various frameshift mutations	ND
Srivastava et al., 2017 [56]	Laboratory-derived mutants	United States	*M. tuberculosis*	*Rv0545c, Rv0930, Rv2477* and *Rv3331, Rv0890c*	Various single-nucleotide variants	>3

ND: Not described. ^ The authors did not describe the DNA mutation. * These two mutations were developed in a second-step mutant and were found together with the *fadD32* mutation. ^#^ Mutations were identified in the genome data of clinical isolates downloaded from databases.

## Data Availability

Not applicable.

## Abbreviations

MIC	Minimum inhibitory concentration
MTB	*Mycobacterium tuberculosis*
NTM	Non-tuberculous mycobacteria
MDR-TB	Multidrug-resistant tuberculosis
PTC	Peptidyltransferase centre
TB	Tuberculosis
XDR-TB	Extensively drug-resistant tuberculosis
WHO	World Health Organisation

## References

[1] Lee M.-R., Sheng W.-H., Hung C.-C., Yu C.-J., Lee L.-N., Hsueh P.-R. (2015). *Mycobacterium Abscessus* Complex Infections in Humans. Emerg. Infect. Dis..

[2] Gygli S.M., Borrell S., Trauner A., Gagneux S. (2017). Antimicrobial Resistance in *Mycobacterium Tuberculosis*: Mechanistic and Evolutionary Perspectives. FEMS Microbiol. Rev..

[3] Saxena S., Spaink H.P., Forn-Cuní G. (2021). Drug Resistance in Nontuberculous Mycobacteria: Mechanisms and Models. Biology.

[4] Rudra P., Hurst-Hess K., Lappierre P., Ghosha P. (2018). High Levels of Intrinsic Tetracycline Resistance in *Mycobacterium Abscessus* Are Conferred by a Tetracycline-Modifying Monooxygenase. Antimicrob. Agents Chemother..

[5] da Silva P.E.A., von Groll A., Martin A., Palomino J.C. (2011). Efflux as a Mechanism for Drug Resistance in *Mycobacterium tuberculosis*. FEMS Immunol. Med. Microbiol..

[6] Palomino J.C., Martin A. (2014). Drug Resistance Mechanisms in *Mycobacterium tuberculosis*. Antibiotics.

[7] Ng H.F., Tan J.L., Zin T., Yap S.F., Ngeow Y.F. (2018). A Mutation in Anti-Sigma Factor MAB_3542c May Be Responsible for Tigecycline Resistance in *Mycobacterium abscessus*. J. Med. Microbiol..

[8] Aw K.M., Ng H.F., Lee C.L., Zin T., Ngeow Y.F. (2022). RshA Mutations Contributing to Tigecycline Resistance in *Mycobacteroides abscessus*. J. Med. Microbiol..

[9] Lee C.L., Ng H.F., Ngeow Y.F., Thaw Z. (2021). A Stop-Gain Mutation in Sigma Factor SigH (MAB_3543c) May Be Associated with Tigecycline Resistance in *Mycobacteroides abscessus*. J. Med. Microbiol..

[10] Ng H.F., Ngeow Y.F. (2022). Genetic Determinants of Tigecycline Resistance in *Mycobacteroides abscessus*. Antibiotics.

[11] Monego F., Duarte R.S., Biondo A.W. (2012). *GyrA* and *GyrB* Gene Mutation in Ciprofloxacin-Resistant *Mycobacterium Massiliense* Clinical Isolates from Southern Brazil. Microb. Drug Resist..

[12] Ng H.F., Ngeow Y.F., Yap S.F., Zin T., Tan J.L. (2020). Tigecycline Resistance May Be Associated with Dysregulated Response to Stress in *Mycobacterium abscessus*. Int. J. Med. Microbiol..

[13] Kochi A., Vareldzis B., Styblo K. (1993). Multidrug-Resistant Tuberculosis and Its Control. Res. Microbiol..

[14] World Health Organisation (2022). Global Tubercolosis Report 2022.

[15] Shah N.S., Wright A., Bai G.-H., Barrera L., Boulahbal F., Martín-Casabona N., Drobniewski F., Gilpin C., Havelková M., Lepe R. (2007). Worldwide Emergence of Extensively Drug-Resistant Tuberculosis. Emerg. Infect. Dis..

[16] WHO Announces Updated Definitions of Extensively Drug-Resistant Tuberculosis. https://www.who.int/news/item/27-01-2021-who-announces-updated-definitions-of-extensively-drug-resistant-tuberculosis.

[17] Han X.Y., Seo Y.H., Sizer K.C., Schoberle T., May G.S., Spencer J.S., Li W., Nair R.G. (2008). A New *Mycobacterium* Species Causing Diffuse Lepromatous Leprosy. Am. J. Clin. Pathol..

[18] Vera-Cabrera L., Escalante-Fuentes W.G., Gomez-Flores M., Ocampo-Candiani J., Busso P., Singh P., Cole S.T. (2011). Case of Diffuse Lepromatous Leprosy Associated with “*Mycobacterium lepromatosis*”. J. Clin. Microbiol..

[19] Han X.Y., Sizer K.C., Thompson E.J., Kabanja J., Li J., Hu P., Gómez-Valero L., Silva F.J. (2009). Comparative Sequence Analysis of *Mycobacterium Leprae* and the New Leprosy-Causing *Mycobacterium lepromatosis*. J. Bacteriol..

[20] Aubry A., Sammarco Rosa P., Chauffour A., Fletcher M.L., Cambau E., Avanzi C. (2022). Drug Resistance in Leprosy: An Update Following 70 Years of Chemotherapy. Infect. Dis. Now..

[21] Worobec S. (2012). Current Approaches and Future Directions in the Treatment of Leprosy. Res. Rep. Trop. Med..

[22] Burgos J., De La Cruz E., Paredes R., Andaya C.R., Gelber R.H. (2011). The Activity of Several Newer Antimicrobials against Logarithmically Multiplying *M. Leprae* in Mice. Lepr. Rev..

[23] Wallace R.J., Dukart G., Brown-Elliott B.A., Griffith D.E., Scerpella E.G., Marshall B. (2014). Clinical Experience in 52 Patients with Tigecycline-Containing Regimens for Salvage Treatment of *Mycobacterium Abscessus* and *Mycobacterium Chelonae* Infections. J. Antimicrob. Chemother..

[24] Sim Y.S., Park H.Y., Jeon K., Suh G.Y., Kwon O.J., Koh W.-J. (2010). Standardized Combination Antibiotic Treatment of *Mycobacterium Avium* Complex Lung Disease. Yonsei Med. J..

[25] Mirsaeidi M., Farshidpour M., Allen M.B., Ebrahimi G., Falkinham J.O. (2014). Highlight on Advances in Nontuberculous Mycobacterial Disease in North America. BioMed. Res. Int..

[26] Moellering R.C. (2003). Linezolid: The First Oxazolidinone Antimicrobial. Ann. Intern. Med..

[27] Swaney S.M., Aoki H., Ganoza M.C., Shinabarger D.L. (1998). The Oxazolidinone Linezolid Inhibits Initiation of Protein Synthesis in Bacteria. Antimicrob. Agents Chemother..

[28] Ippolito J.A., Kanyo Z.F., Wang D., Franceschi F.J., Moore P.B., Steitz T.A., Duffy E.M. (2008). Crystal Structure of the Oxazolidinone Antibiotic Linezolid Bound to the 50S Ribosomal Subunit. J. Med. Chem..

[29] Tsai K., Stojković V., Lee D.J., Young I.D., Szal T., Klepacki D., Vázquez-Laslop N., Mankin A.S., Fraser J.S., Fujimori D.G. (2022). Structural Basis for Context-Specific Inhibition of Translation by Oxazolidinone Antibiotics. Nat. Struct. Mol. Biol..

[30] Henwood C.J. (2000). Susceptibility of Gram-Positive Cocci from 25 UK Hospitals to Antimicrobial Agents Including Linezolid. J. Antimicrob. Chemother..

[31] Behra-Miellet J., Calvet L., Dubreuil L. (2003). Activity of Linezolid against Anaerobic Bacteria. Int. J. Antimicrob. Agents.

[32] Bialvaei A.Z., Rahbar M., Yousefi M., Asgharzadeh M., Kafil H.S. (2017). Linezolid: A Promising Option in the Treatment of Gram-Positives. J. Antimicrob. Chemother..

[33] Schumacher A., Trittler R., Bohnert J.A., Kümmerer K., Pagès J.M., Kern W.V. (2007). Intracellular Accumulation of Linezolid in Escherichia Coli, Citrobacter Freundii and Enterobacter Aerogenes: Role of Enhanced Efflux Pump Activity and Inactivation. J. Antimicrob. Chemother..

[34] Guzel Kaya G., Medaglia S., Candela-Noguera V., Tormo-Mas M.Á., Marcos M.D., Aznar E., Deveci H., Martínez-Máñez R. (2020). Antibacterial Activity of Linezolid against Gram-Negative Bacteria: Utilization of ε-Poly-l-Lysine Capped Silica Xerogel as an Activating Carrier. Pharmaceutics.

[35] Wallace R.J., Brown-Elliott B.A., Ward S.C., Crist C.J., Mann L.B., Wilson R.W. (2001). Activities of Linezolid against Rapidly Growing Mycobacteria. Antimicrob. Agents Chemother..

[36] Chetchotisakd P., Anunnatsiri S. (2014). Linezolid in the Treatment of Disseminated Nontuberculous Mycobacterial Infection in Anti-Interferon-Gamma Autoantibody-Positive Patients. Southeast Asian J. Trop. Med. Public Health.

[37] Zhang X., Falagas M.E., Vardakas K.Z., Wang R., Qin R., Wang J., Liu Y. (2015). Systematic Review and Meta-Analysis of the Efficacy and Safety of Therapy with Linezolid Containing Regimens in the Treatment of Multidrug-Resistant and Extensively Drug-Resistant Tuberculosis. J. Thorac. Dis..

[38] Rapid Communication: Key Changes to the Treatment of Drug-Resistant Tuberculosis. https://www.who.int/publications/i/item/WHO-UCN-TB-2022-2.

[39] Conradie F., Diacon A.H., Ngubane N., Howell P., Everitt D., Crook A.M., Mendel C.M., Egizi E., Moreira J., Timm J. (2020). Treatment of Highly Drug-Resistant Pulmonary Tuberculosis. N. Engl. J. Med..

[40] Bolhuis M.S., Van Der Laan T., Kosterink J.G.W., Van Der Werf T.S., Van Soolingen D., Alffenaar J.W.C. (2014). In Vitro Synergy between Linezolid and Clarithromycin against *Mycobacterium tuberculosis*. Eur. Respir. J..

[41] Zhao W., Zheng M., Wang B., Mu X., Li P., Fu L., Liu S., Guo Z. (2016). Interactions of Linezolid and Second-Line Anti-Tuberculosis Agents against Multidrug-Resistant *Mycobacterium Tuberculosis* in Vitro and in Vivo. Int. J. Infect. Dis..

[42] Williams K.N., Brickner S.J., Stover C.K., Zhu T., Ogden A., Tasneen R., Tyagi S., Grosset J.H., Nuermberger E.L. (2009). Addition of PNU-100480 to First-Line Drugs Shortens the Time Needed to Cure Murine Tuberculosis. Am. J. Respir. Crit. Care Med..

[43] Richter E., Rüsch-Gerdes S., Hillemann D. (2007). First Linezolid-Resistant Clinical Isolates of *Mycobacterium tuberculosis*. Antimicrob. Agents Chemother..

[44] Azimi T., Khoshnood S., Asadi A., Heidary M., Mahmoudi H., Kaviar V.H., Hallajzadeh M., Nasiri M.J. (2022). Linezolid Resistance in Multidrug-Resistant *Mycobacterium Tuberculosis*: A Systematic Review and Meta-Analysis. Front. Pharmacol..

[45] Wasserman S., Louw G., Ramangoaela L., Barber G., Hayes C., Omar S.V., Maartens G., Barry C., Song T., Meintjes G. (2019). Linezolid Resistance in Patients with Drug-Resistant TB and Treatment Failure in South Africa. J. Antimicrob. Chemother..

[46] Tornheim J.A., Intini E., Gupta A., Udwadia Z.F. (2020). Clinical Features Associated with Linezolid Resistance among Multidrug Resistant Tuberculosis Patients at a Tertiary Care Hospital in Mumbai, India. J. Clin. Tuberc. Other Mycobact. Dis..

[47] Ushtanit A., Mikhailova Y., Lyubimova A., Makarova M., Safonova S., Filippov A., Borisov S., Zimenkov D. (2021). Genetic Profile of Linezolid-Resistant *M. Tuberculosis* Clinical Strains from Moscow. Antibiotics.

[48] Broda A., Jebbari H., Beaton K., Mitchell S., Drobniewski F. (2013). Comparative Drug Resistance of *Mycobacterium Abscessus* and *M. Chelonae* Isolates from Patients with and without Cystic Fibrosis in the United Kingdom. J. Clin. Microbiol..

[49] Ye M., Xu L., Zou Y., Li B., Guo Q., Zhang Y., Zhan M., Xu B., Yu F., Zhang Z. (2019). Molecular Analysis of Linezolid-Resistant Clinical Isolates of *Mycobacterium abscessus*. Antimicrob. Agents Chemother..

[50] Yang S.-C., Hsueh P.-R., Lai H.-C., Teng L.-J., Huang L.-M., Chen J.-M., Wang S.-K., Shie D.-C., Ho S.-W., Luh K.-T. (2003). High Prevalence of Antimicrobial Resistance in Rapidly Growing Mycobacteria in Taiwan. Antimicrob. Agents Chemother..

[51] Tu H.Z., Lee H.S., Chen Y.S., Lee S.S.J. (2022). High Rates of Antimicrobial Resistance in Rapidly Growing Mycobacterial Infections in Taiwan. Pathogens.

[52] Nambiar R., Tornheim J., Diricks M., De Bruyne K., Sadani M., Shetty A., Rodrigues C. (2021). Linezolid Resistance in *Mycobacterium Tuberculosis* Isolates at a Tertiary Care Centre in Mumbai, India. Indian J. Med. Res..

[53] Beckert P., Hillemann D., Kohl T.A., Kalinowski J., Richter E., Niemann S., Feuerriegel S. (2012). RplC T460C Identified as a Dominant Mutation in Linezolid-Resistant *Mycobacterium Tuberculosis* Strains. Antimicrob. Agents Chemother..

[54] Ng H.F., Ngeow Y.F. (2023). Mutations in Genes Encoding 23S RRNA and FadD32 May Be Associated with Linezolid Resistance in *Mycobacteroides abscessus*. Microb. Drug Resist..

[55] Li S., Poulton N.C., Chang J.S., Azadian Z.A., Dejesus M.A., Ruecker N., Zimmerman M.D., Eckartt K., Bosch B., Engelhart C. (2021). A Chemical-Genetic Map of the Pathways Controlling Drug Potency in *Mycobacterium tuberculosis*. BioRxiv.

[56] Srivastava S., Magombedze G., Koeuth T., Sherman C., Pasipanodya J.G., Raj P., Wakeland E., Deshpande D., Gumbo T. (2017). Linezolid Dose That Maximizes Sterilizing Effect While Minimizing Toxicity and Resistance Emergence for Tuberculosis. Antimicrob. Agents Chemother..

[57] Long K.S., Vester B. (2012). Resistance to Linezolid Caused by Modifications at Its Binding Site on the Ribosome. Antimicrob. Agents Chemother..

[58] Makafe G.G., Cao Y., Tan Y., Julius M., Liu Z., Wang C., Njire M.M., Cai X., Liu T., Wang B. (2016). Role of the Cys154Arg Substitution in Ribosomal Protein L3 in Oxazolidinone Resistance in *Mycobacterium tuberculosis*. Antimicrob. Agents Chemother..

[59] Klitgaard R.N., Ntokou E., Nørgaard K., Biltoft D., Hansen L.H., Trædholm N.M., Kongsted J., Vester B. (2015). Mutations in the Bacterial Ribosomal Protein L3 and Their Association with Antibiotic Resistance. Antimicrob. Agents Chemother..

[60] Davidovich C., Bashan A., Yonath A. (2008). Structural Basis for Cross-Resistance to Ribosomal PTC Antibiotics. Proc. Natl. Acad. Sci. USA.

[61] Kadura S., King N., Nakhoul M., Zhu H., Theron G., Köser C.U., Farhat M. (2020). Systematic Review of Mutations Associated with Resistance to the New and Repurposed *Mycobacterium Tuberculosis* Drugs Bedaquiline, Clofazimine, Linezolid, Delamanid and Pretomanid. J. Antimicrob. Chemother..

[62] Zhang S., Chen J., Cui P., Shi W., Shi X., Niu H., Chan D., Yew W.W., Zhang W., Zhang Y. (2016). *Mycobacterium Tuberculosis* Mutations Associated with Reduced Susceptibility to Linezolid. Antimicrob. Agents Chemother..

[63] Davies C., Bussiere D.E., Golden B.L., Porter S.J., Ramakrishnan V., White S.W. (1998). Ribosomal Proteins S5 and L6: High-Resolution Crystal Structures and Roles in Protein Synthesis and Antibiotic Resistance. J. Mol. Biol..

[64] Stelzl U., Spahn C.M.T., Nierhaus K.H. (2000). Selecting RRNA Binding Sites for the Ribosomal Proteins L4 and L6 from Randomly Fragmented RRNA: Application of a Method Called SERF. Proc. Natl. Acad. Sci. USA.

[65] Pi R., Liu Q., Jiang Q., Gao Q. (2019). Characterization of Linezolid-Resistance-Associated Mutations in *Mycobacterium Tuberculosis* through WGS. J. Antimicrob. Chemother..

[66] Li S., Poulton N.C., Chang J.S., Azadian Z.A., DeJesus M.A., Ruecker N., Zimmerman M.D., Eckartt K.A., Bosch B., Engelhart C.A. (2022). CRISPRi Chemical Genetics and Comparative Genomics Identify Genes Mediating Drug Potency in *Mycobacterium tuberculosis*. Nat. Microbiol..

[67] LaMarre J.M., Howden B.P., Mankin A.S. (2011). Inactivation of the Indigenous Methyltransferase RlmN in *Staphylococcus Aureus* Increases Linezolid Resistance. Antimicrob. Agents Chemother..

[68] Smith T.M., Jiang Y.-F., Shipley P., Floss H.G. (1995). The Thiostrepton-Resistance-Encoding Gene in *Streptomyces Laurentii* Is Located within a Cluster of Ribosomal Protein Operons. Gene.

[69] Sander P., Belova L., Kidan Y.G., Pfister P., Mankin A.S., Böttger E.C. (2002). Ribosomal and Non-Ribosomal Resistance to Oxazolidinones: Species-Specific Idiosyncrasy of Ribosomal Alterations. Mol. Microbiol..

[70] Carroll P., Faray-Kele M.C., Parish T. (2011). Identifying Vulnerable Pathways in *Mycobacterium Tuberculosis* by Using a Knockdown Approach. Appl. Environ. Microbiol..

[71] Kuhn M.L., Alexander E., Minasov G., Page H.J., Warwrzak Z., Shuvalova L., Flores K.J., Wilson D.J., Shi C., Aldrich C.C. (2016). Structure of the Essential *Mtb* FadD32 Enzyme: A Promising Drug Target for Treating Tuberculosis. ACS Infect. Dis..

[72] Marrakchi H., Lanéelle M.-A., Daffé M. (2014). Mycolic Acids: Structures, Biosynthesis, and Beyond. Chem. Biol..

[73] Ng H.F., Ngeow Y.F. (2020). A Single-Gene Approach for the Subspecies Classification of *Mycobacteroides abscessus*. Pathog. Dis..

[74] Cafini F., Nguyen L.T.T., Higashide M., Román F., Prieto J., Morikawa K. (2016). Horizontal Gene Transmission of the Cfr Gene to MRSA and *Enterococcus*: Role of Staphylococcus Epidermidis as a Reservoir and Alternative Pathway for the Spread of Linezolid Resistance. J. Antimicrob. Chemother..

[75] Nguyen L. (2016). Antibiotic Resistance Mechanisms in *M. Tuberculosis*: An Update. Arch. Toxicol..

[76] Boritsch E.C., Khanna V., Pawlik A., Honoré N., Navas V.H., Ma L., Bouchier C., Seemann T., Supply P., Stinear T.P. (2016). Key Experimental Evidence of Chromosomal DNA Transfer among Selected Tuberculosis-Causing Mycobacteria. Proc. Natl. Acad. Sci. USA.

[77] Parsons L.M., Jankowski C.S., Derbyshire K.M. (1998). Conjugal Transfer of Chromosomal DNA in *Mycobacterium smegmatis*. Mol. Microbiol..

